# Predicting Long-Term Cognitive Outcome Following Breast Cancer with Pre-Treatment Resting State fMRI and Random Forest Machine Learning

**DOI:** 10.3389/fnhum.2017.00555

**Published:** 2017-11-15

**Authors:** Shelli R. Kesler, Arvind Rao, Douglas W. Blayney, Ingrid A. Oakley-Girvan, Meghan Karuturi, Oxana Palesh

**Affiliations:** ^1^Department of Neuro-Oncology, University of Texas MD Anderson Cancer Center, Houston, TX, United States; ^2^Department of Bioinformatics and Computational Biology, University of Texas MD Anderson Cancer Center, Houston, TX, United States; ^3^Division of Medical Oncology, School of Medicine, Stanford University, Palo Alto, CA, United States; ^4^Cancer Prevention Institute of California, Fremont, CA, United States; ^5^Department of Breast Medical Oncology, University of Texas MD Anderson Cancer Center, Houston, TX, United States; ^6^Department of Psychiatry and Behavioral Sciences, School of Medicine, Stanford University, Palo Alto, CA, United States

**Keywords:** breast cancer, chemotherapy, connectome, resting state fMRI, random forest, cognition

## Abstract

We aimed to determine if resting state functional magnetic resonance imaging (fMRI) acquired at pre-treatment baseline could accurately predict breast cancer-related cognitive impairment at long-term follow-up. We evaluated 31 patients with breast cancer (age 34–65) prior to any treatment, post-chemotherapy and 1 year later. Cognitive testing scores were normalized based on data obtained from 43 healthy female controls and then used to categorize patients as impaired or not based on longitudinal changes. We measured clustering coefficient, a measure of local connectivity, by applying graph theory to baseline resting state fMRI and entered these metrics along with relevant patient-related and medical variables into random forest classification. Incidence of cognitive impairment at 1 year follow-up was 55% and was predicted by classification algorithms with up to 100% accuracy (*p* < 0.0001). The neuroimaging-based model was significantly more accurate than a model involving patient-related and medical variables (*p* = 0.005). Hub regions belonging to several distinct functional networks were the most important predictors of cognitive outcome. Characteristics of these hubs indicated potential spread of brain injury from default mode to other networks over time. These findings suggest that resting state fMRI is a promising tool for predicting future cognitive impairment associated with breast cancer. This information could inform treatment decision making by identifying patients at highest risk for long-term cognitive impairment.

## Introduction

Breast cancer and its treatments can have neurotoxic effects in some patients, resulting in acute, persistent and/or late onset cognitive impairments such as difficulties with attention, memory, processing speed, executive function and verbal fluency (Wefel et al., 2015). Defining cognitive impairment has historically been a challenge for cancer-related neurotoxicity as well as other neurologic syndromes. The Diagnostic Statistical Manual-5 (DSM-5) includes a category, “mild neurocognitive disorder” (Saykin et al., 2013; Sachs-Ericsson and Blazer, 2015), which is often used in clinical practice for patients with cancer-related cognitive impairment. Currently there is no diagnostic criteria specific to patients with cancer, however, the International Cognition and Cancer Task Force (ICCTF) recommends an approach for defining cancer-related cognitive impairment (Wefel et al., 2011), which we employed in this study. Clinically, the ICCTF definition corresponds to “mild to moderate impairment”, which is consistent with the DSM-5 criteria for mild neurocognitive disorder and with the level of deficit commonly observed in patients with cancer (Wefel et al., 2015). A previous study observed that the ICCTF definition is almost twice as sensitive to impairment as other methods (Vardy et al., 2017) and we have shown that it corresponds with measures of brain network connectivity (Kesler et al., 2015, 2016).

There are many potential etiologies for cancer-related cognitive impairment, but the final common biologic pathway is altered brain structure and function, which can be evaluated using neuroimaging. In addition to providing valuable insights regarding these neural mechanisms, baseline neuroimaging biomarkers can serve as predictors of future outcome. Several examples of this application exist in other conditions including predicting Alzheimer’s disease conversion, development of dyslexia, and response to interventions such as cognitive rehabilitation (Strangman et al., 2010; Hoeft et al., 2011; Moradi et al., 2015; Thompson et al., 2015). Cancer-related cognitive impairment is one of the only syndromes where a potential brain injury is known in advance and is therefore ideal for prediction of outcome from early or pre-treatment, baseline data. This information could be practice-changing by assisting oncologists with treatment decision-making based on a patient’s individual risk for negative cognitive effects.

Resting state functional magnetic resonance imaging (fMRI), which is one of the most sensitive neuroimaging techniques currently available, is non-invasive and simple to acquire (Kesler, 2014). Resting state fMRI data characterize spontaneous, spatially and temporally coherent functional activity in the brain and are typically used to measure intrinsic functional network connectivity (Raichle, 2011). Intrinsic functional networks reflect various cognitive states, represent the majority of energy usage in the brain and are associated with the expression of genes that regulate synaptic function (Fox and Greicius, 2010; Shirer et al., 2012; Buckner et al., 2013; Richiardi et al., 2015). We previously demonstrated that resting state fMRI data in combination with machine learning can be used to automatically distinguish chemotherapy-treated breast cancer survivors from chemotherapy naïve survivors and healthy female controls (Kesler et al., 2013). Others have shown that resting state fMRI is sensitive to brain networks that recover following chemotherapy vs. those that do not (Dumas et al., 2013).

We recently demonstrated subtle disruption of intrinsic functional network organization in patients newly diagnosed with breast cancer who were evaluated prior to any treatment, including surgery (Kesler et al., 2017a). These findings indicate that resting state fMRI can detect brain changes that are likely associated with aspects of tumor pathology and/or pre-existing patient characteristics that are important for cognitive trajectory. These early neural deficits may make the brain more vulnerable to the effects of chemotherapy, other adjuvant treatments and/or aging, resulting in long-term cognitive impairment.

The application of machine learning algorithms (Jordan and Mitchell, 2015) frequently provides increased prediction accuracy since these models tend to be nonparametric and able to learn complex interactions among predictors. These characteristics are especially important when studying cognition since brain function arises from complex interactions among various neuronal communities. Additionally, machine learning approaches tend to be more suitable than traditional statistical methods for problems such as cancer-related cognitive impairment that involve a large number of potential predictors (Strobl et al., 2007). There are many different machine learning approaches. Random forest modeling uses random subsets of features to grow an ensemble of decision trees that predict the outcome of interest for classification and regression problems (Breiman et al., 1984; Breiman, 2001). We have previously demonstrated that random forest models are highly useful for evaluating cancer-related cognitive impairment (Kesler et al., 2016, 2017b). For example, we showed that random forest models are superior to traditional linear models for examining the relationships between neuroimaging metrics and cancer-related cognitive impairment (Kesler et al., 2016).

In this study, we aimed to predict chronic cognitive impairment (observed at 1 year post-chemotherapy) from baseline intrinsic functional network characteristics obtained prior to treatment initiation. There are many different characteristics of intrinsic functional connectivity that can be measured from resting state fMRI. Based on our prior studies of breast and other cancers (Kesler et al., 2015, 2016, 2017b), we focused on clustered connectivity (i.e., clustering coefficient), a property of brain networks derived from graph theoretical analysis (Rubinov and Sporns, 2010). We examined the accuracy of regional clustering coefficients for predicting categorical cognitive impairment (impaired, unimpaired) by entering them into random forest classification models. We hypothesized that regional clustering coefficients alone or in combination with patient/medical factors would more accurately predict future cognitive outcome than patient/medical factors alone.

## Materials and Methods

### Participants

As part of our ongoing, prospective longitudinal study of breast cancer and cognition, we enrolled 31 newly diagnosed patients with primary breast cancer age 34–65 years and 43 frequency matched healthy control females (Table 1). Patients were assessed prior to initiation of any treatment (including surgery with general anesthesia), 1 month after completing chemotherapy and again 1 year later. Controls were assessed at yoked intervals. Participants were included in the present study if they had completed both the baseline and 1 year follow-up assessments (see Kesler et al., 2017a or Supplementary Methods). Chemotherapy regimens included doxorubicin, cyclophosphamide and paclitaxel (*N* = 16), cyclophosphamide, doxorubicin and fluorouracil (*N* = 2), cyclophosphamide and paclitaxel (*N* = 9), doxorubicin, carboplatin and paclitaxel (*N* = 2), fluorouracil, epirubicin and cyclophosphamide (*N* = 2). The Stanford University Institutional Review Board approved this study and all procedures performed were in accordance with the ethical standards of the Declaration of Helsinki. Written informed consent was obtained from all participants included in the study.

**Table 1:** Participant data at pre-treatment baseline shown as mean (standard deviation) unless otherwise noted.

	Breast cancer (*N* = 31)	Healthy controls (*N* = 43)	*t/X*^2^	*p*
Age	48.58 (8.61)	50.05 (10.1)	0.673	0.503
Age range	34.74–65.73	25.78–64.24		
Education	16.87 (2.86)	17.56 (2.40)	1.08	0.281
Post-menopause	31%	42%	0.357	0.550
Months between 1st and 2nd assessment	5.54 (0.91)	5.30 (0.94)	1.02	0.313
Months between 2nd and 3rd assessment	12.22 (1.68)	12.61 (1.02)	1.03	0.313
Number of chemotherapy cycles	7.25 (4.68)			
Radiation therapy	65%			
Endocrine therapy	71%			
Stage at diagnosis (I, II, III)	16%, 65%, 19%			

### Cognitive Impairment Assessment

Cognitive function was measured using the following standardized tests: Rey Auditory Verbal Learning Test (RAVLT) for verbal learning and verbal memory retention (Schmidt, 2012), Comprehensive Trail Making Test (CTMT) for attention, processing speed and executive function (Moses, 2004), and Controlled Oral Word Association (COWA) for verbal fluency (Ruff et al., 1996). This is consistent with the testing battery recommended by the ICCTF for harmonizing studies of cancer and cognition (Wefel et al., 2011). This is also the battery we have shown previously to be sensitive to cognitive deficits in patients with breast cancer (Kesler and Blayney, 2016; Kesler et al., 2017a). Psychological distress (depression, anxiety, cognitive fatigue) was assessed using the Total Score from the Clinical Assessment of Depression (CAD; Aghakhani and Chan, 2007). We also examined self-ratings from our Mobile Cognitive Assessment Battery Adjustment Index, a questionnaire regarding functional capacity (i.e., occupational, home, leisure and social function (Kesler and Blayney, 2014)). Additional self-report questionnaires, as well as several non-standardized, experimental computerized tests, were administered but are not reported here (total testing time = 1.5 h).

Test scores were converted to *z*-scores based on the control group’s mean and standard deviation. Cognitive impairment was defined as having any two *z*-scores of −1.5 or lower or any one *z*-score of −2.0 or lower, based on the ICCTF recommendations (Wefel et al., 2011) and our prior studies (Kesler et al., 2015, 2016, 2017b). A patient was categorized as impaired if her performance was impaired at both baseline and 1 year follow-up (persistent impairment) or if she demonstrated impaired performance at 1 year follow-up that was not present at baseline (late onset impairment). As noted above, we have previously demonstrated that this impairment definition is associated with measures of brain network organization. Impairment was also moderately associated with elevated symptoms on the Adjustment Index (*r* = 0.29, *p* = 0.059), suggesting further ecological validity.

### Neuroimaging Acquisition and Preprocessing

Neuroimaging data were acquired using a GE Discovery MR750 3.0 Tesla whole body scanner (GE Medical Systems) on the same day as the cognitive testing session (see Supplementary Methods for further details). Functional connectivity preprocessing was performed with Statistical Parametric Mapping 8 (SPM8) and CONN Toolboxes as previously described (Kesler et al., 2013, 2014, 2017a; Kesler and Blayney, 2016). The resulting connectivity matrices were binarized to minimum connection density and submitted to graph theoretical analysis using our Brain Networks Toolbox^1^. As in our previous studies, 90 regions of interest (ROIs) were defined using the Automated Anatomical Labeling Atlas (Tzourio-Mazoyer et al., 2002) and we measured the clustering coefficient of each brain ROI. Clustering coefficient is the ratio of connections to all possible connections among a region’s neighbors (Rubinov and Sporns, 2010). We have previously demonstrated significant clustering deficits in patients with breast and other cancers (Bruno et al., 2012; Hosseini et al., 2012; Kesler et al., 2015, 2016).

### Statistical Analysis

Incidence of cognitive impairment was compared between groups using a two-sample test for equality of proportions (Chi squared, two-tailed). Change in CAD and Adjustment Index scores were evaluated using paired *t*-test.

For random forest classification, the square root of the number of features were split at each node and an ensemble of 500 trees was grown by bootstrapping the features with replacement. Feature selection/reduction was conducted on a training set (A + B) consisting of a 75% random sample of the breast cancer group obtained after stratified class sampling. Recursive feature elimination was used to remove minimally contributing features and optimize the models. Recursive feature elimination was conducted on this set with A = training data and B = testing data with leave-one-out cross-validation across 100 random partitions of A and B. Features that provided the best accuracy across these partitions were used to re-train a model on A + B with out-of-bag error estimation (Liaw and Wiener, 2002). The resulting model was then applied to the held-out 25% of the breast cancer group to test prediction accuracy.

**Figure 1:**
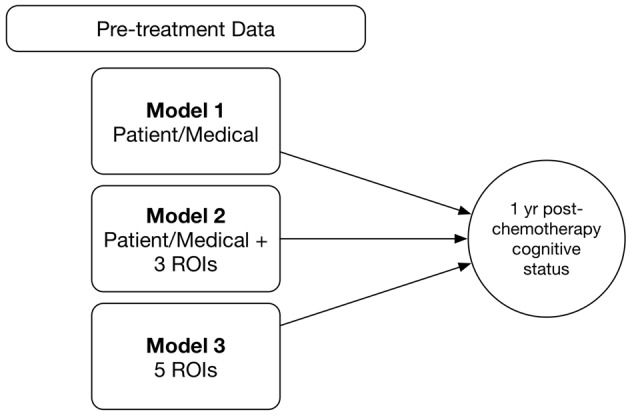
Random forest models. We tested and compared three different random forest models for predicting 1 year post-chemotherapy cognitive outcome from pre-treatment data. ROIs, connectome regions of interest.

We tested three different models (Figure 1). Model 1 included only the following baseline patient and medical features: age, education, cancer stage at diagnosis, minority status, menopausal status and CAD score. Model 2 combined the above patient/medical features with clustering coefficients for three brain regions; right middle orbitofrontal gyrus, right inferior parietal lobule (RIPL) and right mesial superior frontal gyrus. We previously showed these regions to have subtly altered clustering prior to treatment in patients with breast cancer (Kesler et al., 2017a). For Model 3, we tested an expanded feature set that included clustering coefficients for all major cortical and subcortical regions (*N* = 90) in addition to patient/medical features.

The significance of model accuracy was evaluated using a two-sided exact binomial test in addition to the area under the curve (AUC) of the receiver operating characteristic (ROC). Feature importance was determined using mean decrease in Gini index (Wright et al., 2016; Kesler et al., 2017b). To determine the most accurate model, we compared model AUCs using the bootstrapping method described by Hanley and McNeil (1983).

Brain regions identified as important predictors were evaluated for network hub status based on degree, betweenness centrality and/or clustering coefficient greater than 1 standard deviation above network mean (Sporns et al., 2007). We also evaluated modularity to provide insight regarding hub relationships. Modularity involves decomposing the brain into non-overlapping groups of regions (modules) that have maximal within-group connections and minimal between-group connections (Sporns and Betzel, 2016). Hubs were further classified as provincial or connector type based on module participation coefficient per previously established criteria (Guimerà and Amaral, 2005; Sporns et al., 2007).

Because there is no standard definition of cognitive impairment, we supplemented classification analysis with random forest regression to determine if features identified by classification could accurately predict individual cognitive test *z*-scores at 1 year follow-up. Regression model accuracy was determined using the adjusted R squared statistic. Feature importance for regression models was determined using percent increase in mean squared error. All statistical analyses were performed in the R Statistical Package (R Foundation) including the “randomForest”, “caret” and “pROC” libraries.

## Results

### Cognitive Impairment

Patients with breast cancer demonstrated 55% (*N* = 17/31) incidence of cognitive impairment and healthy controls demonstrated 26% (*N* = 11/43). The difference in incidence was significant (*X*^2^ = 6.56, *p* = 0.010). Of those impaired in the breast cancer group, 59% (*N* = 10/17) had persistent impairment while 41% (*N* = 7/17) had late onset impairment. Depression, anxiety and fatigue decreased over time based on patients’ self-ratings, but not significantly (*p* > 0.725) and was not clinically elevated at any time point. Self-rating of functional capacity decreased over time but not significantly (*p* > 0.193). Cognitive testing and self-report data are presented in Table 2 and Supplementary Table S1.

**Table 2:** Cognitive testing *z*-scores for patients with breast cancer.

Test name	Mean (standard deviation)/range		
	Pre-treatment *N* = 31	Post-chemotherapy *N* = 23	1 year post-chemotherapy *N* = 31
RAVLT A1	−0.452 (1.00)	−0.326 (0.928)	−0.428 (0.944)
	−2.92 to 1.28	−2.10 to 1.70	−1.99 to 1.21
RAVLT A6	−0.329 (1.40)	−0.227 (0.836)	−0.308 (1.08)
	−3.07 to 1.58	−1.48 to 1.28	−3.33 to 1.30
CTMT 1	−0.469 (0.842)	−0.454 (0.952)	−0.478 (1.01)
	−2.68 to 1.17	−1.94 to 0.966	−2.10 to 2.30
CTMT 5	−0.251 (0.894)	−0.322 (1.12)	−0.421 (1.07)
	−1.70 to 1.13	−2.31 to 1.37	−2.08 to 1.41
COWA	−0.314 (0.904)	−0.434 (0.844)	−0.040 (0.707)
	−2.15 to 1.55	−1.96 to 1.11	−1.79 to 1.21

*RAVLT, Rey Auditory Verbal Learning Test; CTMT, Comprehensive Trail Making Test; COWA, Controlled Oral Word Association*.

### Predicting Future Cognitive Impairment

**Figure 2:**
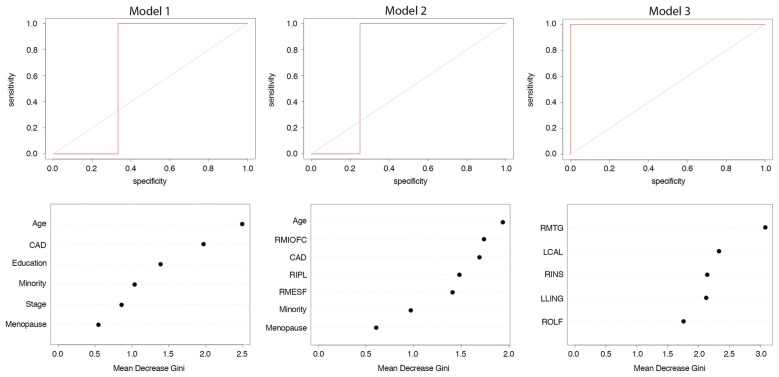
Random forest classification of cognitive impairment at 1 year post-chemotherapy follow-up from pre-treatment predictors. Model 1: patient and medical variables; Model 2: clustering coefficients from *a priori* brain regions and patient/medical variables; Model 3: clustering coefficients from 90 brain regions and patient/medical variables. Top row shows receiver operating characteristic (ROC) curve. Bottom row shows relative feature importance. Features displayed are those retained after recursive feature elimination. Higher mean decrease in Gini index indicates greater importance of that feature in the model. CAD, Clinical Assessment of Depression; RMIOFC, right middle orbitofrontal cortex; RIPL, right inferior parietal; RMESF, right mesial superior frontal; RMTG, right middle temporal; LCAL, left calcarine; RINS, right insula; LLING, left lingual; ROLF, right olfactory.

Model 1: the final model retained all six features and performed with 71% accuracy (*p* = 0.453, ROC = 0.67, sensitivity = 0.75, specificity = 0.67, Figure 2). Model 2: the final model retained the three brain regions as well as age, minority status, menopausal status and CAD and demonstrated 88% accuracy (*p* = 0.070, ROC = 0.75, sensitivity = 1.0, specificity = 0.75, Figure 2). Model 3: the final model retained five brain regions (left lingual gyrus, left calcarine, right insula, right middle temporal gyrus and right olfactory area) but no patient/medical features and performed with 100% accuracy (*p* = 0.008, ROC = 1.0, sensitivity = 1.0, specificity = 1.0, Figure 2).

### Comparing Classification Models

The AUC of Model 3 was significantly greater than the AUCs of Models 1 (*z* = 2.83, *p* = 0.005) and 2 (*z* = 2.42, *p* = 0.015). Models 1 and 2 were not significantly different (*z* = 0.514, *p* = 0.607).

### Predicting Future Cognitive Test Scores

Using the five brain regions from Model 3 above, individual RAVLT verbal learning scores were accurately predicted with an adjusted *R*^2^ = 0.79 (*p* < 0.0001). RAVLT verbal retention scores were predicted with an adjusted *R*^2^ = 0.78 (*p* < 0.0001). The model for CTMT Trail 1 scores had an adjusted *R*^2^ = 0.70 (*p* < 0.0001). CTMT Trail 5 scores were predicted at adjusted *R*^2^ = 0.75 (*p* < 0.0001). The model for COWA scores had an adjusted *R*^2^ = 0.64 (*p* < 0.0001). The relative contributions of the five clustered connectivity features to these regression models are provided in Supplementary Table S2.

### Characteristics of Predictive Brain Regions

**Figure 3:**
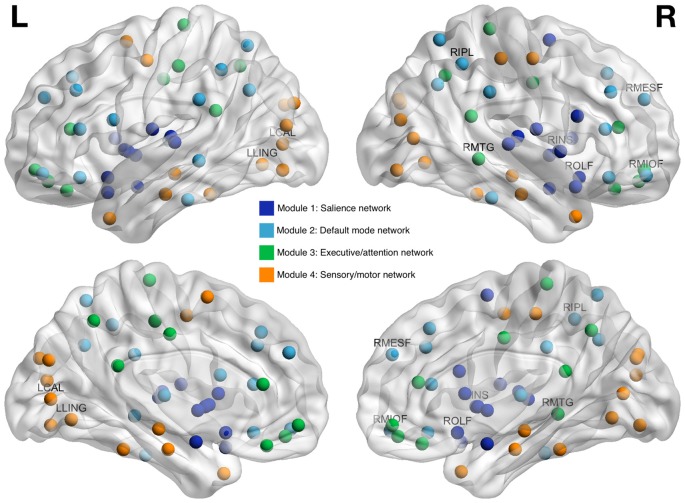
Brain regions whose clustering coefficients at pre-treatment predicted cognitive impairment at 1 year post-chemotherapy follow-up. Regions are shown as spheres with color indicating module membership. Labeled regions are those that were included in random forest classification models and found to be predictive of cognitive impairment. RMIOF, right middle orbitofrontal; RIPL, right inferior parietal; RMESF, right mesial superior frontal; RMTG, right middle temporal; LCAL, left calcarine; RINS, right insula; LLING, left lingual; ROLF, right olfactory.

As shown in Table 3, all but two of the eight brain regions included in Models 2 and 3 were categorized as hubs (e.g., globally connected regions) and all were connector type hubs. Modularity analysis indicated that all regions from Model 2 were in the default mode network while regions from Model 3 were members of other networks including sensory/motor, executive/attention and salience networks (Figure 3).

**Table 3:** Hub characterization for brain regions that predicted cognitive impairment.

Region	Degree	Betweenness	Clustering	Participation coefficient	Hub	Hub type
Left calcarine	19.81	444	0.54	0.47	No	-
Right middle orbitofrontal	15.13	803	0.64*	0.53	Yes	Connector
Right mesial superior frontal	20.13	1042*	0.50	0.58	Yes	Connector
Right insula	19.77	998*	0.59	0.54	Yes	Connector
Left lingual	21.03	615	0.53	0.39	No	-
Right olfactory	15.26	732	0.60*	0.59	Yes	Connector
Right inferior parietal	15.42	1077*	0.55	0.44	Yes	Connector
Right middle temporal	24.48*	563	0.52	0.64	Yes	Connector

*Denotes values that exceeded 1 standard deviation above the network mean and thus indicate hub status. Connector hubs are defined as those with participation coefficient greater than 0.3 and provincial hubs have a participation coefficient less than 0.3.

## Discussion

The aim of this study was to determine if baseline, pre-treatment resting state fMRI could be used to accurately predict long-term cognitive outcome in chemotherapy-treated patients with breast cancer. Based on our prior work, we measured clustering coefficient, a characteristic of brain network connectivity obtained from resting state fMRI data. We observed that most patients (55%) demonstrated cognitive impairment at 1 year post-chemotherapy follow-up. This incidence is consistent with previous studies (Wefel et al., 2010, 2015). We examined three different classification models for predicting this impairment: Model 1 included only patient/medical variables, Model 2 combined patient/medical variables with clustering coefficients from selected, *a priori* regions, and Model 3 included the entire brain with patient/medical variables.

Model 1 results indicated that patient and medical factors, particularly age and CAD score, were independently useful at predicting impairment with 71% accuracy, although specificity was suboptimal and the overall model was not significant. The addition of clustered connectivity data improved accuracy to 85% with increased specificity, though this improvement was not significant. Model 3 performed with perfect sensitivity and specificity and included only clustered connectivity of brain regions selected in a data driven manner. Model 3 was significantly more accurate than Models 1 and 2. Further, regression models using Model 3 features were associated with significant adjusted R^2^ values for predicting individual test scores suggesting that the model may be relatively robust to impairment definition. Perfect (100%) accuracy in machine learning applications is rare but not unprecedented when neuroimaging features are included (Gothelf et al., 2011; Marzelli et al., 2011; Weygandt et al., 2012; Zhang et al., 2013). However, our model was built using a small sample and therefore requires subsequent validation.

Many of the regions selected in Models 2 and 3 have been noted to be altered in prior studies of breast cancer as well as other conditions that affect cognitive function (Kaiser et al., 2014; Kesler, 2014; Lepage et al., 2014; Stouten-Kemperman et al., 2015; Wang et al., 2016). These regions are known to be associated with the cognitive domains we measured. For example, orbitofrontal regions are involved in executive control and other cognitive processes (Nestor et al., 2015; Ohtani et al., 2017). The insula is a key region of the salience network, important for various functions including attention, language, interoception and social-emotional behaviors (Menon and Uddin, 2010; Seeley, 2010). Middle temporal gyrus supports memory, language, semantic and visual processing, among others, and is part of the ventral attention network (Deslauriers et al., 2017). One novel finding was the importance of the right olfactory area. Olfaction has a well-known and important role in memory through conditional and emotional learning systems (Mouly and Sullivan, 2010) and as a site of ongoing adult neurogenesis (Lledo and Valley, 2016). Cancer treatments, including chemotherapy and radiation interfere with neurogenesis (Monje and Dietrich, 2012) and have been associated with changes in olfactory function in patients with breast and other cancers (Steinbach et al., 2010).

Modularity analysis indicated that all Model 2 regions were part of the default mode network, consistent with prior studies (Fox et al., 2005; Seeley et al., 2007; Grayson and Fair, 2017). Default mode network subserves a wide variety of cognitive processes and is therefore characterized by high connectivity and functional activity (Hagmann et al., 2008; Cole et al., 2010; Lord et al., 2013). The “nodal stress” theory of neurodegeneration suggests that high traffic regions, like hubs of the default mode network, are more vulnerable to aging, disease and injury (Zhou et al., 2012). Our findings suggest that certain default mode network hubs are injured by breast cancer and this injury does not adequately recover over time and/or is exacerbated by adjuvant therapies such that it is associated with long-term cognitive impairment.

The regions in Model 3 most accurately predicted outcome but had no overlap with those in Model 2; we did not previously observe them to be different between patients with breast cancer and healthy controls at pre-treatment baseline (Kesler et al., 2017a). Whereas Model 2 regions were members of default mode network, Model 3 regions were included in salience, executive/attention and sensory/motor networks. These regions may be very subtly vulnerable pre-treatment such that differences are difficult to detect and/or alternative methods are required to detect them. Otherwise, default mode network injury existing at pre-treatment baseline might extend to other brain networks via “trans-neuronal spread” (Zhou et al., 2012). The brain regions included in Model 2 were all identified as hubs; regions with high connectivity that are vital for network resilience and regulation of information flow (Vertes and Bullmore, 2015). Further, Model 2 regions were all connector type hubs, which, unlike provincial type hubs, form bridges between different networks (van den Heuvel and Sporns, 2013). Taken together, these findings suggest that the initial site of injury involves default mode network hubs that potentially spread the injury to other networks via their connector status. Most Model 3 regions were also identified as hubs and as such would be the most vulnerable areas of these “secondary” networks. These mechanisms were not the primary focus of this study and therefore further investigation of longitudinal changes in connectome organization is required.

The main limitation of this study is the small sample size which can result in model over-fitting. We conducted random forest modeling using a conservative approach that included cross-validation and careful separation of training and testing samples. However, further evaluation of our models’ validity requires a new, unseen and larger sample of patients to which we can apply our algorithms. We are currently acquiring such a sample and have also made our algorithms available for others to apply to their own data as appropriate^2^. Other considerations include our choice of brain parcellation scheme and connectome property. The 90 AAL parcellation is one of the most commonly used and is the one we have employed previously in our studies of chemotherapy-related cognitive impairment (Kesler et al., 2015, 2016, 2017a,b; Amidi et al., 2017). As noted above, we focused on clustering coefficient because we have shown this connectome property to be the most consistently altered in patients with breast cancer. Future studies with larger samples could include evaluation of alternative connectome properties to determine if they improve predictive models. These might include connectome properties derived from other neuroimaging modalities such as diffusion tensor imaging (DTI), for example. We used a cognitive testing battery and impairment definition recommended by the ICCTF to increase consistency across studies of cancer and cognition including data pooling initiatives. Further investigation is required to examine the effects of alternate tests and impairment categories. Finally, other machine learning approaches may yield different results. For example, support vector machine (SVM) is a common method used in neuroimaging studies and we have previously demonstrated its usefulness for distinguishing chemotherapy-treated from chemotherapy naïve patients (Kesler et al., 2013). However, SVMs are much more difficult to interpret than random forest models, particularly in terms of feature importance. Feature importance contributed to evaluation of our hypothesis regarding relative importance of patient/medical vs. neuroimaging features and was essential for understanding specific brain network patterns involved in cognitive impairment. Future studies could include comparison of different machine learning approaches, which was beyond the scope of this preliminary study.

Patients undergoing chemotherapy tend to be monitored for various toxicities including cardiac, hepatic and hematologic problems, among others. Given the high incidence of cognitive impairments, it seems reasonable that neurologic monitoring be included as well. We have demonstrated that this impairment can potentially be predicted from baseline, pre-treatment data. Resting state fMRI may be a particularly promising tool for this purpose, improving our ability to identify patients at risk for long-term cancer-related brain injury. If inclusion of resting state fMRI data continues to result in the most accurate predictions of future cognitive outcome, we have already demonstrated that it is feasible to obtain these data from patients pre-treatment. Connectome metrics derived from resting state fMRI show good to excellent test-retest reliability (Braun et al., 2012; Cao et al., 2014; Termenon et al., 2016). Our resting state fMRI acquisition required only 7 min making this scan a practical possibility. Prediction of cognitive outcome could inform treatment decision-making and prioritize patients for early intervention. With further validation, our findings could support the use of one of our algorithms as standard of care for patients with breast cancer to determine risk for cognitive neurotoxicity.

## Author Contributions

SRK designed the study, wrote the Matlab code for graph theoretical analysis and the R code for random forest analysis, conducted the statistical analyses and prepared the manuscript. AR assisted with R code and statistical analyses and edited the manuscript. IAO-G and MK edited the manuscript. DWB provided oncology consultation, assisted with participant recruitment and edited the manuscript. OP supervised participant recruitment, study coordination and data acquisition and edited the manuscript.

## Conflict of Interest Statement

The authors declare that the research was conducted in the absence of any commercial or financial relationships that could be construed as a potential conflict of interest.

## Footnotes

^1^https://github.com/srkesler/bnets.git

^2^https://www.dropbox.com/sh/df3akk7wr4wl2uv/AACYvBdZnnThXLqT-3OQgJxla?dl=0
