# Supplementary material for: Predicting Long-Term Cognitive Outcome Following Breast Cancer with Pre-Treatment Resting State fMRI and Random Forest Machine Learning
Source: Front Hum Neurosci. 2017 Nov 15;11:555. doi: 10.3389/fnhum.2017.00555 (PMC5694825; doi:10.3389/fnhum.2017.00555)
Supplement: Supplementary file 1 [file Table_1.docx]

**Predicting long-term cognitive outcome following breast cancer with pre-treatment resting state fMRI and random forest machine learning**

Shelli R. Kesler, PhD, Arvind Rao, PhD, Douglas W. Blayney, MD, Ingrid Oakley-Girvan, PhD, MPH, Meghan Karuturi, MD, Oxana Palesh, PhD, MPH

**Supplementary Methods**

Patients with breast cancer were recruited at the Stanford Cancer Institute. All consecutive patients who met study eligibility criteria were approached. Healthy controls were recruited via local media advertisements in northern California communities. There were 156 participants screened; 32 were excluded or declined to participate. Participants were excluded for psychiatric, neurologic, or comorbid medical conditions that are known to affect cognitive function as well as any major sensory deficits (e.g., blindness). Participants were also required to be fluent in English sufficient for valid cognitive testing.

Resting state fMRI was obtained while participants rested in the scanner with their eyes closed. We used a T2*-weighted gradient echo spiral pulse sequence ^1^ with the following parameters: relaxation time = 2000 msec, echo time = 30 msec, flip angle = 89° and 1 interleave, field of view = 200, matrix = 64x64, in-plane resolution = 3.125. Number of volumes collected was 216, scan time = 7:12. An automated high-order shimming method was used to reduce field inhomogeneity. We also acquired a high-resolution, 3D inversion-recovery prepared fast spoiled gradient echo T1-weighted anatomical MRI scan with the following parameters: TR = minimum, TE = minimum, flip = 11 degrees, inversion time = 300 msec, bandwidth = +/-31.25 kHz, field of view = 24cm, phase field of view = 0.75, slice thickness = 1.5mm, 125 slices, 256x256 at 1 excitation, scan time = 4:26. This volume was used only for spatial normalization of resting state fMRI. Some participants also underwent diffusion tensor imaging if time allowed (total scan time = 30 minutes or less). These data are not reported here. Neuroimaging data were visually inspected for quality prior to preprocessing and none required exclusion.

**Supplementary Table S1.** Cognitive testing and self-report questionnaire data for patients with breast cancer and healthy controls shown as mean (standard deviation).

| Test Name | Breast cancer | | | Healthy female controls | | |
| --- | --- | --- | --- | --- | --- | --- |
|  | Time 1  N = 31 | Time 2  N = 23 | Time 3  N = 31 | Time 1  N = 43 | Time 2  N = 39 | Time 3  N = 43 |
| RAVLT A1 | 53 (7.7) | 54 (7.6) | 54 (8.3) | 56 (7.9) | 57 (8.0) | 57 (8.8) |
| RAVLT A6 | 11 (3.0) | 11 (2.1) | 10 (3.0) | 12 (2.2) | 12 (2.6) | 11 (2.8) |
| CTMT 1 | 51 (8.1) | 56 (11) | 53 (9.7) | 55 (9.5) | 62 (12) | 58 (9.6) |
| CTMT 5 | 52 (8.5) | 54 (9.4) | 53 (9.5) | 54 (9.8) | 57 (8.7) | 57 (8.9) |
| COWA | 46 (12) | 45 (12) | 47 (9.0) | 49 (13) | 51 (15) | 52 (13) |
| CAD | 53 (9.4) | 52 (8.6) | 51 (10) | 43 (8.6) | 44 (10) | 44 (11) |
| MCAB Adjustment Index | 1.2 (1.5) | 1.7 (1.5) | 1.7 (1.9) | 0.67 (0.88) | 0.61 (1.0) | 0.73 (1.2) |

RAVLT: Rey Auditory Verbal Learning Test (raw scores), CTMT: Comprehensive Trail Making Test (T-scores), COWA: Controlled Oral Word Association (adjusted raw scores); CAD: Clinical Assessment of Depression (T-scores); MCAB: Mobile Cognitive Assessment Battery (raw scores). Higher CAD or MCAB scores = elevated symptoms/problems whereas higher scores on all other tests = better performance. CAD scores of 69 or higher indicate clinically significant psychological distress.

Time 1: pre-treament; Time 2: post-chemotherapy; Time 3: 1 year post-chemotherapy or yoked intervals for controls

**Supplementary Table S2.** Feature importance for random forest regression models. Data show percent increase in mean squared error for permuted (randomized) values of each feature. Higher values correspond to greater importance of that feature in the model (i.e. mean squared error increases if the feature value is random).

|  | Left calcarine | Left lingual | Right insula | Right middle temporal | Right olfactory |
| --- | --- | --- | --- | --- | --- |
| RAVLT A1 | 0.025 | -0.013 | 0.096 | -0.013 | 0.114 |
| RAVLT A6 | 0.020 | -0.074 | 0.266 | -0.015 | 0.155 |
| CTMT 1 | -0.034 | -0.022 | -0.048 | 0.048 | 0.196 |
| CTMT 5 | 0.004 | 0.117 | -0.091 | 0.161 | 0.094 |
| COWA | -0.065 | -0.036 | -0.011 | -0.029 | 0.012 |

RAVLT: Rey Auditory Verbal Learning Test, CTMT: Comprehensive Trail Making Test, COWA: Controlled Oral Word Association

1 Glover, G. H. & Law, C. S. Spiral-in/out BOLD fMRI for increased SNR and reduced susceptibility artifacts. *Magn Reson Med* **46**, 515-522 (2001).
